# Nutritional Education in Medical Curricula and Clinical Practice: A Scoping Review on the Knowledge Deficit Amongst Medical Students and Doctors

**DOI:** 10.1111/jhn.70031

**Published:** 2025-03-06

**Authors:** Nasr Khiri, Kristy Howells

**Affiliations:** ^1^ Kent and Medway Medical School Canterbury UK; ^2^ Canterbury Christ Church University Canterbury UK

**Keywords:** knowledge deficit, medical curricula, medical students and doctors, nutrition education

## Abstract

**Introduction:**

Non‐communicable diseases (NCDs), accounting for 74% of deaths worldwide (World Health Organization 2024), are a major health concern and are often the result of poor dietary habits. To reduce the prevalence of chronic diseases healthcare professionals must encourage healthy eating, and therefore require the appropriate nutritional knowledge and skills. This scoping review critically synthesises the literature on nutrition education to understand why there is a gap in nutrition knowledge and skills among medical students and doctors (MSAD) in English‐speaking countries, and the solutions which have been proposed in the literature to close this gap.

**Methods:**

This scoping review adhered to PRISMA Scr guidelines outlined by Tricco et al. (2018) and used four online databases: PubMed; WebOfScience; Embase and ERIC as well as grey literature sources: Google; Bing and Perplexity AI, published within the last 10 years, from 2014 to 2024. Studies investigating medical students/doctors nutrition education/knowledge were included. Data analysis was guided by Braun and Clarke's (2012) six‐step thematic analysis approach and the Delve qualitative coding software analysis tool was used to identify the two principal themes and the 20 sub‐themes. The PICO tool was also used for question analysis.

**Results:**

From the 674 records identified, 28 papers met the inclusion criteria for full data extraction, analysis and synthesis. The results identified four reasons for the gap in nutrition knowledge, including insufficient curriculum time dedicated to nutrition education, perceptions and confidence, stigmas and health habits, and challenges in clinical practice. The review also identified four potential solutions to minimise this gap, including curriculum changes, enforcement of standardised nutrition education guidelines, integration of nutrition in clinical practice and promotion of a multidisciplinary approach to nutrition education.

**Conclusion:**

This scoping review shows that there are multiple complex reasons for the gap in nutrition knowledge and understanding. This is due to education institutional reasons; perceptions and confidence on nutrition; stigmas related to nutrition and being able to talk about nutrition; personal health habits; and challenges in clinical practice. Solutions to reduce the gap were identified and it is recommended that there are curricular innovations to incorporate nutrition education throughout medical training; standardisation and implementation of national competency standards; integration of nutrition into clinical practice; enhancement of postgraduate education pathways in nutrition; and the development of a multidisciplinary approach in medical education involving dietitians and other healthcare professionals. By enacting and supporting the recommendations and solutions would then improve patient care and likely contribute to better eating habits worldwide, thereby reducing the burden of NCDs to both patients and healthcare professionals.

## Summary

- The knowledge deficit amongst Medical Students And Doctors (MSAD) arises from perceptions and confidence levels, habits, stigmas and challenges in clinical practice.
- The results from the scoping review suggest the gaps can be addressed through curriculum innovation, national competency standards, clinical integration, enhanced postgraduate pathways and multidisciplinary collaboration.
- The benefits of implementing the recommended solutions will be seen in improvements in patient care, by fostering healthier eating habits and by impacting on NCDs.

## 1 Introduction

Good nutrition is the cornerstone of the prevention and management of most non‐communicable diseases (NCDs), such as diabetes, cardiovascular disease and obesity. Although poor diets, often due to inadequate nutrition, contribute to 74% of global deaths from NCDs [1], nutrition has rarely been given much attention in the curricula for healthcare professionals. Patel and Kassam [2] identified that ‘most medical schools do not sufficiently teach their students the clinical application of nutrition science’ (p. 861) This paper reviews the gaps in knowledge and competencies on nutrition among medical students and doctors (MSAD) and discusses the shortcomings of the current medical curriculum in providing adequate knowledge for future and current practitioners to meet the nutritional needs of their patients effectively.

Healthy eating can prevent the onset of NCDs: diets rich in fruits and vegetables, which are high in dietary fibre, can reduce the risk of obesity, cardiovascular disease and type 2 diabetes, while antioxidants found in many fruits and vegetables can lower the risk of cancer [3]. Providing the body with the necessary nutrients to function optimally reduces the likelihood of developing poor mental and physical health conditions, and improves the quality of life [4]. Asher et al. [5] emphasised that ‘poor diet, including inadequate vegetable intake is one of the leading risk factors’ for NCD (p. 967). To reduce the prevalence of chronic diseases healthcare professionals must encourage healthy eating, and they require the appropriate nutritional knowledge and skills. Medical students and professionals must be equipped with adequate nutrition knowledge and skills.

Crowley et al. [6] found that educators and students agreed on the importance of incorporating more nutrition education into medical curricula. As a result, the authors suggested that the inclusion of multidisciplinary cooperation, hands‐on training opportunities, and integrating nutrition education into the current curricula would improve overall nutrition knowledge and understanding.

Although nutrition education plays an important role in equipping healthcare professionals to effectively tackle the rising prevalence of NCDs, several critical deficiencies persist in MSAD's training. According to Lepre et al. [7], systemic barriers include a lack of adequate training and institutional support. Jones et al. [8, 9] similarly discuss missed opportunities in nutrition integration into basic medical sciences such as biochemistry and physiology. Such training gaps mean that future practitioners will be ill‐prepared to respond effectively to the dietary needs of their patients.

This scoping review investigates why medical curricula in English‐speaking countries inadequately prepare students to address patient's dietary needs and explores solutions to address this gap. The subsequent sections are structured as follows: first, the review outlines and justifies the methodology used. Second, it presents the research findings by dividing the discussion into two parts: the first part examines the reasons for the lack of nutrition education in medical curricula, and the second part examines solutions which have been identified by studies to fill this gap. The ultimate objective of this scoping review is to increase nutrition education received by MSADs to improve patient care and increase healthy eating habits worldwide.

## 2 Materials and Methods

This scoping review adhered to the PRISMA SCr guidelines outlined by Tricco et al. [10]. From 2 October 2023 to 25 February 2024, the relevant literature was searched across four different databases: PubMed, WebOfScience, Embase, and ERIC. Search strings included: ‘medical students’, ‘doctors’ or ‘physicians’ and ‘nutritional knowledge’, ‘nutrition education’ or ‘nutrition training’ and relevant Boolean operators to ensure a broad search. Table 1 lists the search terms and hits from each database.

**Table 1:** Data bases, search terms, hits and the number selected.

Database	Search terms	Hits	Selected
PubMed	(medical students* OR student doctors* OR medical undergraduates* OR doctors* OR physicians*) AND (nutritional knowledge* OR nutrition education* OR nutritional awareness* OR nutrition training* OR diet education* OR food education* OR food knowledge* OR food education*) AND (United Kingdom* OR England* OR Wales* OR Northern Ireland* OR Scotland* OR USA* OR CANADA* OR AUSTRALIA* OR NEW ZEALAND*)	160	16 (12)
WebOfScience	(medical students* OR student doctors* OR medical undergraduates* OR doctors* OR physicians*) AND (nutritional knowledge* OR nutrition education* OR nutritional awareness* OR nutrition training* OR diet education* OR food education* OR food knowledge* OR food education*) AND (United Kingdom* OR England* OR Wales* OR Northern Ireland* OR Scotland* OR USA* OR CANADA* OR AUSTRALIA* OR NEW ZEALAND*)	410	25 (17)
Embase (Ovid)	(medical students* OR student doctors* OR medical undergraduates* OR doctors* OR physicians*) AND (nutrition knowledge* OR nutrition education* OR nutritional awareness* OR nutrition training* OR diet education* OR food education* OR food knowledge* OR food education*) AND (United Kingdom* OR England* OR Wales* OR Northern Ireland* OR Scotland* OR United States of America* OR CANADA* OR AUSTRALIA* OR NEW ZEALAND*)	67	10
ERIC	(medical students* OR student doctors* OR medical undergraduates* OR doctors* OR physicians*) AND (nutritional knowledge* OR nutrition education* OR nutritional awareness* OR nutrition training* OR diet education* OR food education* OR food knowledge* OR food education*) AND (United Kingdom* OR England* OR Wales* OR Northern Ireland* OR Scotland* OR United States of America* OR CANADA* OR AUSTRALIA* OR NEW ZEALAND*)	20	6

*Note:* Also, a grey literature search was conducted using Google and Bing, using the key search phrase: ‘nutrition knowledge and education in MSADs in English‐speaking countries’.

The eligibility criteria are outlined in Table 2 (inclusion) and Table 3 (exclusion).

**Table 2:** Inclusion criteria.

Inclusion criteria	Rationale
Articles published within the last 10 years	To ensure in date literature
Studies focusing on medical students'/doctors nutrition education and/or knowledge	To ensure relevant data are used.
Peer‐reviewed papers	To ensure data with high credibility are used.

**Table 3:** Exclusion criteria.

Exclusion criteria	Rationale
Non‐English language studies	Translation could lead to misinterpretation and/or errors
Studies focusing on other health professionals other than medical students/doctors	To maintain relevancy to the topic
Non‐peer‐reviewed papers, opinion pieces, letters to the editor, commentaries and abstracts	These do not hold enough credibility to be used

In terms of the evidence source selection process. The 5 part process was followed as set out in Table 4.

**Table 4:** Process of source selection.

Evidence source selection process	
1.	Search results were initially screened against the eligibility criteria, using title and abstract alone.
2.	A full‐text review was conducted for articles where the information provided in the title and abstract was inadequate to determine the study's eligibility for inclusion.
3.	Snowballing techniques—including the examination of the reference list of the chosen articles—were used to identify further studies which were appropriate for inclusion.
4.	Once all appropriate studies had been selected, duplicates were removed manually.
5.	All relevant full‐text scripts were analysed and eligible studies were kept. The remaining studies were then included for narrative synthesis, critical appraisal, data extraction and analysis.

**Figure 1:**
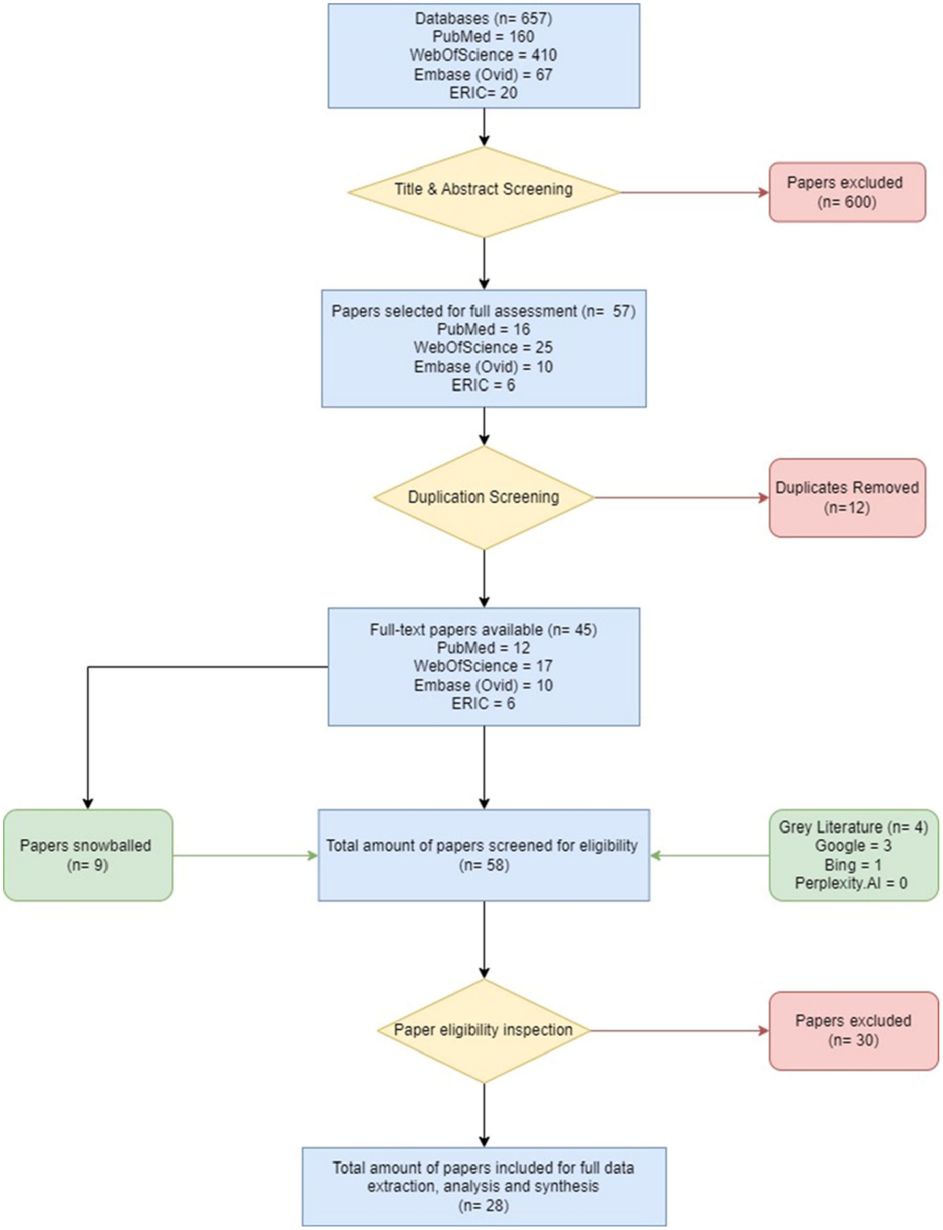
Flowchart of the selection process.

Data were critically appraised using CASP Qualitative Studies Checklist tool [11] with nonrelevant answers excluded. All data critically appraised were put into an excel spreadsheet. The following flowchart (Figure 1) demonstrates how the articles used in the selected, from the initial number of articles found within the database searching, the process of source selection and filtration. As well as the inclusion of papers sourced through grey literature and snowballing.

Data were extracted on the following: year of publication, author, study type, population, research focus, country, assessment method, if they found potential strategies that could enhance nutrition education for MSADs, reasons why there was a lack of nutrition knowledge/awareness in MSADs, if the article was being funded, the papers' outcomes and its limitations. When relevant information was retrieved, then it was put into an excel spreadsheet. If a paper did not answer a question it was left with unapplicable. A thematic analysis following Braun and Clarke [12] six‐step guidance synthesised the results (see Table 5).

**Table 5:** Thematic analysis process.

Thematic analysis six‐step guidance	
1.	The lead researcher familiarised themself with the objectives of this scoping review. Then the lead researcher familiarised themselves with data by reading all the papers.
2.	When data found matched with the objectives the lead researcher created codes to highlight similar groups of data.
3.	From these codes, subthemes began to appear and then themes began to generate.
4.	These themes were then reorganised, edited or removed.
5.	The themes were then refined and named
6.	A discussion of analysis was created using the themes.

## 3 Results

The peer‐reviewed papers were read and analysed. Information that was deemed relevant to the objectives of the scoping review were put into codes. These codes were then grouped to create subthemes, and then finally the two major themes arose. Twenty‐eight peer‐reviewed papers in this study were thematically analysed using Delve's coding system to find insights and to nest and merge codes. Two critical issues/themes were identified during the thematic analysis: first, factors behind the nutritional knowledge deficit in MSADs (Table 6) and second, possible solutions to address this deficit (Table 7).

**Table 6:** Factors behind the nutritional knowledge deficit in MSADs.

Educational reasons	Insufficient time allocated to nutrition education
	Lack of financial resources and specialised educators
	Lack of comprehensive guidelines
	Lack of postgraduate education
Perceptions and Confidence	Nutrition perceived as insignificant
	Low confidence in counselling patients
Stigmas and Health habits	Personal health habits
	Nutrition is a sensitive topic
Challenges in clinical practice	Lack of time, funding and guidance in clinical practice
	The role of dietitians and low rates of referrals

**Table 7:** Possible solutions to address the knowledge deficit in MSADs.

Curricular innovations	Altering the current medical curriculum
	Introducing nutrition into mandatory assessments
	Introducing pre‐requisites for medical school admission
Standardisation and guidelines	Implementing national competency standards for future doctors
Clinical practice integration	Allocating more time and resources for nutrition education and practice within the clinical setting
	Further research into nutrition education in medical education and clinical settings
Professional development and collaboration	Providing more postgraduate education pathways in nutrition
	Developing a multidisciplinary approach in medical education

## 4 Discussion

Four key factors behind the knowledge deficit were educational gaps, perceptions, stigma and clinical practice challenges. These will be in turn each be discussed in more detail.

### 4.1 Factors

#### 4.1.1 Education Reasons

##### 4.1.1.1 Insufficient Time Allocated to Nutrition Education

Most UK medical students report receiving less than 2 h of nutrition education [8, 13]. This lack of time dedicated to nutrition education is like that found within USA. Krishnan et al. [14] most recently reported that 75% of medical schools had no required clinical nutrition classes. Whilst in Canada there is a set minimum number of hours dedicated to nutrition education, but only 27% of the 105 medical schools met this requirement of 25 h, the researchers [15] also found that guidance for patients with obesity was out of date. Yet general practitioners across the global are often the first point of call when patients are struggling with their diet health. The public (in the United Kingdom) may be shocked that the person they are turning to for help and guidance has only had on average less than 2 h focused study on this area. According to Kris‐Etherton et al. [16], there is ‘limited penetration of nutrition into curricula because of competition for time’. Because medical curricula prioritise other topics, less time is available and thus allocated towards nutrition Crowley et al. [17] cite ‘curriculum crowding, scarcity of nutrition advocates, and specialist teachers’ as some of the key reasons for the lack of time allocated towards nutrition education. Limited specialised resources and curriculum prioritisation reduce time for nutrition education.

##### 4.1.1.2 Lack of Financial Resources and Specialised Educators

Blunt and Kafatos [18] find that ‘there are few trained trainers available’ to provide MSADs with nutrition education. Without enough qualified professionals to teach nutrition, medical students are less likely to receive optimal education in this area. Also, a lack of appropriate financial compensation may deter nutrition professionals from participating in teaching nutrition, increasing the shortage of specialised educators, and therefore reducing the quality of nutrition education. According to Burch et al. [19], dietitians who volunteered their time to teach nutrition to students were not financially compensated. The lack of financial compensation has resulted in an insufficient number of nutrition educators. Having fewer trainers also restricts the ability for curriculum development to reflect the latest research and best practices in nutrition, thereby limiting medical students' ability to have the most up‐to‐date knowledge of novel nutritional science.

##### 4.1.1.3 Lack of Comprehensive Guidelines

Ball et al. [20] state that there is a ‘lack of consensus on the best way to develop and deliver nutrition education within medical training’, and that it is up to the medical school's discretion to integrate nutrition. Crowley et al. [21] note that the lack of mandatory enforcement leads to inconsistent nutrition education across medical schools, creating knowledge disparities. Furthermore, Ganis and Christides [13] find that there is no published data on the current outcomes of nutrition‐related education in UK postgraduate training programmes. The lack of curricular guidelines for postgraduate programmes means that there is a missed opportunity for mandatory training/assessments for doctors. This reveals a gap in curricula and a failure to prioritise nutrition education.

Crowley et al. [21] find that a lack of mandatory enforcement is the principal reason for no national nutrition curriculum in English‐speaking countries. This demonstrates inconsistent implementation of nutrition education due to the absence of mandatory enforcement. Ganis and Christides [13] find that even though nutrition objectives have been integrated into curricula because nutrition is not recognised as its own clinical specialty, the objectives in curricula are often disjointed and disorganised. The lack of consistency across medical curricula in terms of nutrition education and training suggests that it is not as significant as other topics.

##### 4.1.1.4 Lack of Postgraduate Education

The Royal Colleges are professional bodies responsible for overseeing postgraduate medical education in the UK. According to Ganis and Christides [13], ‘deficiencies in nutrition‐related education across UK postgraduate medical training have been identified for over 20 years’. They continue and suggest that there may be a systematic failure to integrate nutrition into postgraduate education programmes [13]. Krishnan et al. [14] also concur and found similar deficiencies in postgraduate provision in the USA and advocate for more formal clinical nutrition training. Blunt and Kafatos [18] earlier find that in the United Kingdom ‘there are no avenues governed or provided by any of the royal colleges through which physicians can obtain approved medical training in nutrition’. Blunt and Kafatos [18] concur, noting no structured pathways for doctors to pursue medical nutrition training through the Royal Colleges. The lack of pathways for obtaining approved postgraduate nutritional training shows, again, that nutrition education is not prioritised for medical students.

#### 4.1.2 Perceptions and Confidence

##### 4.1.2.1 Nutrition Perceived as Insignificant

Lennon and Muir [22] found a disconnect between students' perceived and actual relevance of nutrition. The view from medical students indicates a misunderstanding about the role which nutrition plays in preventive health, patient care and the management of the NDCs. Spencer et al. [23] previously reported that the importance of nutrition declined as medical students progressed through their training. This highlights that although medical students initially think that nutrition is highly relevant unless this is reinforced within medical education, a decline can occur in the medical students' perception. Burch et al. [19] also claim that students perceive nutrition as less important than other topics. Lepre, Mansfield, and Beck [24] state that this viewpoint is shared internationally. This perception and attitude towards nutrition education might be the result of a curriculum which prioritises topics that focus on biochemistry and pharmacology. The misperception underlies the lack of nutrition knowledge in healthcare services. MSADs who believe that nutrition education is not significant will not increase the time and funding for nutrition education, nor will they attempt to change the medical curricula and clinical practice to increase focus on this area.

##### 4.1.2.2 Low of Knowledge and Low Confidence to Counsel Patients

Lack of knowledge among practitioners reduces their confidence in offering nutritional advice. Crowley, Ball, and Hiddink [6] find that general practitioners' low motivation is a key reason for why there is a gap in practice. The lack of nutrition knowledge amongst medical practitioners may be the result of inadequate training in nutrition, perceived ineffectiveness of dietary interventions, and/or the challenges of changing patient behaviours. It could also be the result of time pressures in clinical practice and/or prioritisation of acute medical issues. Regardless, the lower the motivation to learn about and practice nutrition the less likely it will be implemented in medical curricula.

Crowley, Ball, and Hiddink [17] find that most medical students lack confidence in counselling patients, this may be due to the paucity of nutrition knowledge within training. Devries et al. [25] similarly found in the USA that MSAD ‘consistently reported insufficient nutrition training and a uniformly low level of confidence’ (p. 465). The lack of confidence to be able to counsel patients can be directly attributed to the inadequate focus on nutrition education. It can also lead to a reluctance to engage in nutritional counselling, reducing nutrition's significance of application in clinical practice, in terms of meaningful discussions with patients, and ultimately further widening the nutrition knowledge gap.

#### 4.1.3 Stigmas and Health Habits

##### 4.1.3.1 Personal Health Habits

Blunt and Kafatos [18] find that doctors are more likely to offer advice if they practice healthy eating habits themselves and similarly, that their counsel is more likely to be readily accepted by patients. Advice from ‘slimmer’ doctors is often given more significance (2019, p. 346). Also, Crowley, Ball, and Hiddink [6] state that GPs with healthy habits provided nutrition care very often in practice. The correlation between doctors’ health habits and the likelihood that they will offer guidance suggests there is a need to provide medical students with nutrition education so that they can engage in healthy lifestyles which is more likely to translate into their practice.

##### 4.1.3.2 Sensitive Topic

Nutrition is perceived as a sensitive topic. There is a tendency, therefore, to avoid discussion about nutrition, diet and eating habits in education and clinical practice. Medical students in Lennon and Muir [22] believed that it would be inappropriate for them to address issues such as weight and diet to patients. Ganis and Christides [13] find that weight stigmatisation is prevalent in medical practice. The stigma surrounding nutrition not only impacts the quality of patient care but also contributes to the nutrition knowledge deficit among medical professionals. Avoiding discussions of nutrition limits practitioners’ opportunities to apply their knowledge, widening the knowledge gap.

#### 4.1.4 Challenges in Clinical Practice

##### 4.1.4.1 Lack of Time, Funding and Guidance in Clinical Practice

According to Murphy et al. [26], oncologists may not be able to identify those at nutritional risk because of the lack of standardised protocols for care. The lack of clinical guidelines emphasises the lack of support for doctors in integrating nutrition into their practice. Anderson and Nguyen [27] report the lack of time as a significant barrier to giving advice to patients’ diets and weights. This is supported by Lepre, Mansfield, and Beck [24], who find that the average GP consultation in the United Kingdom and Australia is 10 and 15 min, respectively. These time constraints show that physicians are under considerable pressure to address complex health issues. Consequently, providing comprehensive care to patients in appointments is less achievable, and thus doctors are less likely to provide nutritional counselling. In addition, Crowley, Ball, and Hiddink [6] identified multiple studies that revealed that primary care physicians were not financially compensated for providing nutrition care. This devalues nutrition's role in patient care but also discourages practitioners from dedicating time and resources to it. This economic disincentive contributes to the deprioritisation of nutrition, further widening the gap in nutritional knowledge and practice among doctors.

##### 4.1.4.2 The Role of Dietitians and Low Rates of Referrals

Burch et al. [19] find that students had not been exposed to the role of dietitians in medical school, and therefore had inaccurate perceptions and assumptions about their roles and relationships with dietitians in clinical practice. Ray et al. [28] found that junior doctors did not make referrals because they did not know how or when to make these referrals to dietitians. The disconnect in understanding the role of dietitians and how they should deal with referrals contributes to ineffective nutritional patient care and low rates of referrals.

The results also found that there were four main solutions that could address the nutritional knowledge deficit in MSADs, these included curricular innovations; standardisation and guidelines; clinical practice integration; and professional development and collaboration. These will be in turn each be discussed in more detail.

### 4.2 Solutions

#### 4.2.1 Curricular Innovations

##### 4.2.1.1 Altering the Current Medical Curriculum

Ball et al. [20] reflect on an initiative at the University of Cambridge in which a vertical spiral curriculum was introduced so that students could develop their nutrition competencies and build on them every year as they progress through their degree. This ensures that nutrition education is not merely a single, isolated component of their training but that it is a part of their entire education. Because the curriculum revisits and expands on nutrition concepts each year, students are able to gradually build upon their existing knowledge. This process solidifies understanding and retention. It also allows the integration of nutrition into different specialties, allowing students to see the relevance of nutrition across the medical practice. This model also introduces practical aspects of learning in clinical years. In the paper, students in their first clinical year have opportunities to speak to patients with obesity. The objective is to lessen the stigma surrounding weight, so that students can have open discussions about the importance and relevance of nutrition. Likewise, Pang et al. [29] and Magallanes et al. [30] advocate for the integration of practical teaching methods to improve medical students' nutrition knowledge. Cooking lessons, for example, teach students to prepare healthy meals and understand the nutritional value of various foods, such an idea of cooking has been previously recommended as a novel teaching method by Patel and Kassam [2]. These methods are likely to increase students' personal health habits and equip students with the confidence to provide dietary advice to future patients. Jones et al. [9] also propose the use of the hidden curriculum not just ‘demonstrating how nutrition can be integrated into clinical practice’ (p. 7) but also how nutrition education has the potential to be implemented in a range of other parts of the curriculum, these could biochemistry, and physiology. It is important though that the medical educators link across the curriculum and use a ‘nutritional science lens’ [9, p. 6].

##### 4.2.1.2 Introducing Nutrition Into Mandatory Assessments

Crowley et al. [21], Xie et al. [31], Ball et al. [20], Nawaz et al. [32] and Jones et al. [9] recommend including nutrition‐related questions in mandatory written and practical assessments. For the written exams, it was proposed that these could include case studies that require an understanding of the nutritional management of chronic diseases, the role of diet in prevention and the interpretation of nutritional research. Similarly, Objective Structured Clinical Examinations (OSCEs) can be used to assess students' competencies in dietary assessment, nutrition counselling, the ability to develop personalised nutrition plans and communication skills. As well alongside the exams and assessments Erlich [33] recommends a three‐part learning model that she termed a ‘3‐course meal’ which comprised of knowledge, application and assessment, with a pre‐class online learning course, then an in‐class learning and then primary care clinical setting learning. These proposed ideas could allow students to acquire sufficient nutritional knowledge and interpersonal/clinical skills to effectively counsel their patients on lifestyle changes.

##### 4.2.1.3 Introducing Pre‐Requisites for Medical School Admission

The Nutrition Academic Award (NAA) experience in van Horn et al. [34] explains that taking an introductory nutrition course before medical school improves nutrition knowledge among medical students. This early exposure prepares students for more advanced topics in nutrition and serves as a strong predictor of academic success, as evidenced by higher USMLE scores in van Horn et al. [34]. This is further supported by Dr. Kohlmeier's proposal in Kris‐Etherton et al. [16] for medical schools to include a nutrition and behaviour modification course as a requirement for admission. This guarantees that all incoming students possess a baseline understanding of nutrition, which can be built upon with more specialised knowledge during medical school teaching.

#### 4.2.2 Standardisation and Guidelines

##### 4.2.2.1 Implementing National Competency Standards for Future Doctors

The Association for Nutrition (AfN), with support from the Academy of Medical Royal Colleges (AoMRC) and the General Medical Council (GMC) has launched an initiative which aims to incorporate nutrition into undergraduate curricula in medical schools in the United Kingdom. As a result of this initiative, 13 core nutritional competencies have been developed, which are assessed through 11 graduation criteria (the details are outlined in AfN [35]). The 13 competencies are broken down into Knowledge, Assessment and Skills in Intervention. The six knowledge competencies emphasise a comprehensive understanding of healthy balanced diets and the inclusion of ethical, legal considerations and collaboration with other healthcare professionals. This ensures future doctors are well versed in theoretical knowledge of nutritional science, while highlighting a holistic approach to nutrition care, recognising it as a complex, multidisciplinary field.

The four assessment competencies include evaluating nutritional status of patients, for example, measuring body mass indexes (BMI). This ensures students attribute greater importance to nutrition education because mandatory assessments can act as powerful motivators for students to engage and learn. Developing practical intervention skills such as initiating sensitive conversations about nutrition and making appropriate referrals, equips medical students with the interpersonal and clinical skills and is necessary for effective patient care. Lastly, the 11 graduation fundamentals serve as a benchmark for what every medical student should achieve by the end of their undergraduate training so this means that future doctors across the UK will share a common foundation in their understanding of nutritional science. Other countries can elevate nutrition in medical education by nationally standardised competencies for future doctors. This has been recommended previously by Patel and Kassam [2] who focused on the idea of developing learning outcomes within the nutrition education curricula to link to national guidance to help standardise content and make the teaching purposeful and applicable.

Nutrition competencies have most recently within the USA, Eisenberg, Cole, Maile, et al. [36] proposed nutrition competencies for both medical students and physical trainees. The authors developed a consensus statement through a modified Delphi process and recommend that at both undergraduate and graduate levels in medical education, there be 36 nutrition competencies and for this to be part of the licensing process and examinations. They also noted that by receiving enhanced nutrition education may enable behaviour change in the MASD and this can then be reflected in increased confidence in counselling patients.

#### 4.2.3 Clinical Practice Integration

##### 4.2.3.1 Allocating More Time and Resources for Nutrition Education and Practice Within the Clinical Setting

To increase the time and resources allocated towards nutrition, GP clinics might need to be designed such as to include dedicated slots for nutrition counselling for patients. This approach is advocated by the Royal Australian College of General Practitioners (RACGP) and the British Medical Association (BMA) in Lepre, Mansfield, and Beck [24]. Through the extension of consultation times, not only is patient care improved but physicians have the opportunity to apply their theoretical knowledge into practice, bridging the gap between medical school teaching and patient care. This method of practice reinforces their nutrition knowledge. The same paper also suggests increased funding via the government as a solution, highlighting the desire for institutional support in making nutrition education a more integral part of medical practice.

##### 4.2.3.2 Further Research Into Nutrition Education in Medical Education and Clinical Settings

Further research is required to address the nutrition knowledge gap in MSADs. According to van Horn et al. [34], there is a need to research physician interventions, educational designs and new strategies to improve doctors’ clinical encounters with nutrition. Similarly, Crowley, Ball, and Hiddink [17] recommend the need for further studies to measure how well nutrition education initiatives align with global recommendations for a healthy diet, such as the EAT‐Lancet Commission. This ensures that medical students have the appropriate knowledge to combat nutrition‐related health challenges in future practice. There is a call for more research to evaluate and improve different parts of nutrition education, from student assessment methods and curriculum delivery to the effectiveness of these curricula in imparting necessary knowledge and skills. This agenda addresses the current voids in empirical evidence. It also attempts to pioneer and test new strategies for teaching nutrition. This goal not only improves the quality of nutrition education but also ensures that future doctors are better prepared to integrate nutrition into their clinical practice, thereby enhancing patient care and public health outcomes.

#### 4.2.4 Professional Development and Collaboration

##### 4.2.4.1 Providing More Postgraduate Education Pathways in Nutrition

Innovative training approaches, such as the lifestyle medicine curriculum in Nawaz et al. [32] that teaches key skills like motivational interviewing and exercise prescriptions, demonstrate the practical routes in which medics can integrate nutrition into their practice. Moreover, the creation of new subspecialties in nutrition and obesity as proposed by the ACGME in van Horn et al. [34] highlights a possible structural advancement in medical education, aiming to produce doctor nutrition subspecialists. These doctors would be able to improve nutrition and obesity‐related patient care in their respective specialty, for example, paediatrics. This training standardises nutrition knowledge across the medical profession, addressing specialist‐level gaps.

##### 4.2.4.2 Developing a Multidisciplinary Approach in Medical Education

The findings of this scoping review have highlighted that the systemic barrier, as identified by Lepre et al. [7], should be addressed through curriculum innovations and a multidisciplinary approach. For example, embedding dietitians into teaching modules and embedding nutrition into existing medical sciences will create a cohesive and practical learning environment for medical students.

Dietitians possess the necessary knowledge and skills to effectively deliver nutrition education within the healthcare setting [37]. Therefore, it is reasonable to suggest that dietitians should lead nutrition teaching along with other senior academics in clinical and public health nutrition to medical students. These teachers can help address inadequate nutrition training as cited by many medical students [17]. Through direct engagement and practical learning, students can better understand nutrition in patient care [28]. These professionals should have an input in the curriculum development of undergraduate medicine to include sufficient nutrition education to medical students. Increased dietitian involvement in teaching and clinical rotations could clarify their role for medical students. Another solution proposed by Xie et al. [31] is to introduce teaching from a multiprofessional team where medical students can have opportunities to understand the different roles and responsibilities of each healthcare professional. This understanding could help to increase more (appropriate) referrals from doctors in the future.

Jones et al. [8] have explained that integration into basic medical sciences such as biochemistry and physiology is necessary, of which metabolic pathways form the routes connecting to clinically prevalent conditions like cardiovascular disorders and diabetes. They further elaborate how integrating nutrition in currently existing models for teaching in programmes such as pharmacology and public health courses offers added advantages in presenting the multidisciplinary needs against the inadequacy of nutrition education. This paper supports the inclusion of a well‐rounded curriculum that incorporates theoretical knowledge, such as biochemistry, into more clinical medicine, such as counselling of patients. In fact, a dietitian–clinician co‐teacher model would explicitly address many of these identified limitations to provide a grounding in and application of nutrition education.

## 5 Conclusion

This scoping review has provided a broad map for both established and emerging researchers in nutrition education within medical curricula. Whereas most of the literature usually discusses a few problems or solutions at a time, this paper synthesises a wide array of literature uniquely to give a holistic view of the persistent gaps in knowledge and practice alongside a wide array of proposed solutions that have emerged over recent years. This extensive compilation is invaluable to the field, in that it offers a clear overview of both the enduring challenges and the progressive advancements within the domain, thereby functioning as an essential framework for guiding future research and curriculum development.

The review has highlighted a significant gap in nutritional knowledge among MSADs due to factors such as insufficient education, lack of resources, and inadequate emphasis on nutrition in curricula and practice. It was determined that the underlying reason was the perceived insignificance of nutrition among MSADs. The scoping review has identified a systemic failure to integrate comprehensive nutrition education at both undergraduate and postgraduate levels. Solutions proposed included curricular innovations, increased postgraduate pathways in nutrition and a multidisciplinary approach involving dietitians. Overall, these findings emphasise the need to improve nutrition education to increase preventive health measures to improve patient care.

The relevance of these findings from the scoping review to professional practice is crucial. Increasing time, resources, and significance of nutrition could improve future medical professionals' approach to patient care. This would also help the need to upskill medical students from their initial deficit and lack of healthy lifestyles, and health education, arisen from school‐based settings. Emphasising preventive health and dietary interventions will reduce NCDs and be more cost effective for patients and professionals. This comprehensive strategy towards nutrition education promises a healthier future for the broader community, emphasising the pivotal role of nutrition in preventive medicine and public health.

## Author Contributions

**Nasr Khiri:** conceptualisation (lead), data curation (lead), formal analysis (lead), investigation (lead), methodology (lead), visualisation (supporting), writing – original draft preparation (lead), writing – review and editing (supporting). **Kristy Howells:** conceptualisation (supporting), supervision (lead), visualisation (lead), writing – original draft preparation (supporting), writing – review and editing (lead).

## Ethics Statement

The scoping review did not require NHS ethics approval, as it does not involve any patient data or interventions. Ethical approval was applied for and gained through Kent and Medway Medical School, Research Ethics Advisory Group, approved 9th May 2023.

## Conflicts of Interest

The authors declare no conflicts of interest.

## Data Availability

The authors have nothing to report.
